# I spy with my little eye: a simple behavioral assay to test color sensitivity on digital displays

**DOI:** 10.1242/bio.035725

**Published:** 2018-08-20

**Authors:** Alexander G. Knorr, Céline M. Gravot, Clayton Gordy, Stefan Glasauer, Hans Straka

**Affiliations:** 1 Center for Sensorimotor Research, Department of Neurology, University Hospital Großhadern, Feodor-Lynen-Str. 19, 81377 Munich, Germany; 2 Department of Biology II, Ludwig-Maximilians-University Munich, Großhaderner Str. 2, 82152 Planegg, Germany; 3 Graduate School of Systemic Neurosciences, Ludwig-Maximilians-University Munich, Großhaderner Str. 2, 82152 Planegg, Germany; 4 Computational Neuroscience, Institute of Medical Technology, Brandenburg University of Technology Cottbus-Senftenberg, Universitätsplatz 1, 01968 Senftenberg, Germany

**Keywords:** Vision, Optokinetic reflex, Eye motion, Amphibian, Virtual reality

## Abstract

Passive and interactive virtual reality (VR) environments are becoming increasingly popular in the field of behavioral neuroscience. While the technique was originally developed for human observers, corresponding applications have been adopted for the research of visual-driven behavior and neural circuits in animals. RGB color reproduction using red, green and blue primary color pixels is generally calibrated for humans, questioning if the distinct parameters are also readily transferable to other species. In particular, a visual image in the RGB color space has a clearly defined contrast pattern for humans, but this may not necessarily be the case for other mammals or even non-mammalian species, thereby impairing any interpretation of color-related behavioral or neuronal results. Here, we present a simple method to estimate the sensitivity of animals to the three primary colors of digital display devices based on the performance of object motion-driven visuo-motor reflexes and demonstrate differences in the color sensitivity between *Xenopus laevis* and *Ambystoma mexicanum* (Axolotl).

This article has an associated First Person interview with the first author of the paper.

## Abstract (precis)

**Summary:** We present a simple method to estimate an animals’ sensitivity to component colors of RGB images based on visuo-motor reflexes and demonstrate its applicability in *Xenopus laevis* and *Ambystoma mexicanum*.

## INTRODUCTION

Over the past two decades, digitally produced visual images for virtual reality (VR) have undergone remarkable technical advancements. Originally designed for military training purposes and video gaming technologies, VR has found its entrance into neuroscientific research (Tarr and Warren, 2002; Thurley and Ayaz, 2017) where it became indispensable for many applications such as navigation research or exposure therapies for phobias (Bohil et al., 2011). VR enables experimenters to implement and imitate complex natural or artificial visual conditions. There are no limitations by the actual laboratory layout, and even scenarios that are impossible in the real world can be emulated, thereby opening new approaches to study human perception and behavior (Tarr and Warren, 2002). It also comes as no surprise that VR setups are more and more employed as powerful tools in animal research as well (Thurley and Ayaz, 2017). Driven by advances in computer processing power and technology, visual virtual environments become constantly more sophisticated and almost indistinguishable from natural scenes (Bastuğ et al., 2017).

Virtual environments can be either interactive or passive and are therefore employed in a closed- or open-loop mode (Roussou and Slater, 2017), with the latter largely serving to present moving or still images for the analysis of visual processing in, for example, humans (Foerster et al., 2016) or for activation of optomotor behavior in zebrafish (Filosa et al., 2016) or amphibian tadpoles (Gravot et al., 2017). Typically, visual scenes are presented by available digital display technologies, such as LCD monitors and image projectors (Buttussi and Chittaro, 2018). These digital devices reproduce natural colors by mixing three distinct component colors (red, green and blue; as defined by the International Telecommunication Union in their Recommendation; see reference list ITU-R BT.601-7). The exact ratio between the three primary colors was determined earlier through intensive psychophysical experimentation in humans (Guild, 1932). The color impression therefore appears realistic to the human eye, although the frequency spectrum of composite colors often differs from real, natural colors (Gegenfurtner and Kiper, 2003). However, since different animal species have different combinations of photoreceptor subtypes, it is unclear and in fact not very likely that the natural color reproduction for digitally generated visual environments for humans is readily transferable to those of other mammalian and even non-mammalian species (D'Eath, 1998; Fleishman et al., 1998). Despite the technical advancements over the last years, relatively little is known about how animals perceive and behaviorally respond to color stimuli that were presented on digital monitors. The digital presentation of realistic natural world-like environments is therefore a challenge since a particular species might perceive an image presented on an RGB display differently compared to real world images (Chouinard-Thuly et al., 2017). Previous efforts towards an adequate calibration of digital display devices have mostly relied on detailed models of retinal photoreceptor sensitivity in the species used for experimentation, however, without actual behavioral verification (Tedore and Johnsen, 2017).

Here, we describe a simple method for estimating the relative sensitivities to the three component colors presented on an RGB display as defined in the software as RGB triplets. The relative sensitivity to each of the component colors was estimated from the performance of contrast-dependent visuo-motor responses to large-field visual image motion. In addition to known methods of measuring behavioral sensitivities to colored visual stimuli (Tedore and Johnsen, 2017; Chouinard-Thuly et al., 2017), we propose a novel approach that allows the experimenter to directly determine the sensitivity to colors in the RGB color space used in the experimental software, without requiring any knowledge about photometric properties of the display, ambient lighting conditions, or retinal photoreceptor sensitivity. The applicability of this method was tested by using rotational motion stimuli to evaluate species-specific differences in color sensitivity of the optokinetic reflex in larvae of the amphibians *Xenopus laevis* and *Ambystoma mexicanum* (Axolotl). This method can be easily extended to other visuomotor behaviors in vertebrates as well as invertebrates by using different visual motion stimuli (e.g. looming stimuli for collision avoidance behavior).

## RESULTS AND DISCUSSION

**Fig. 1.**
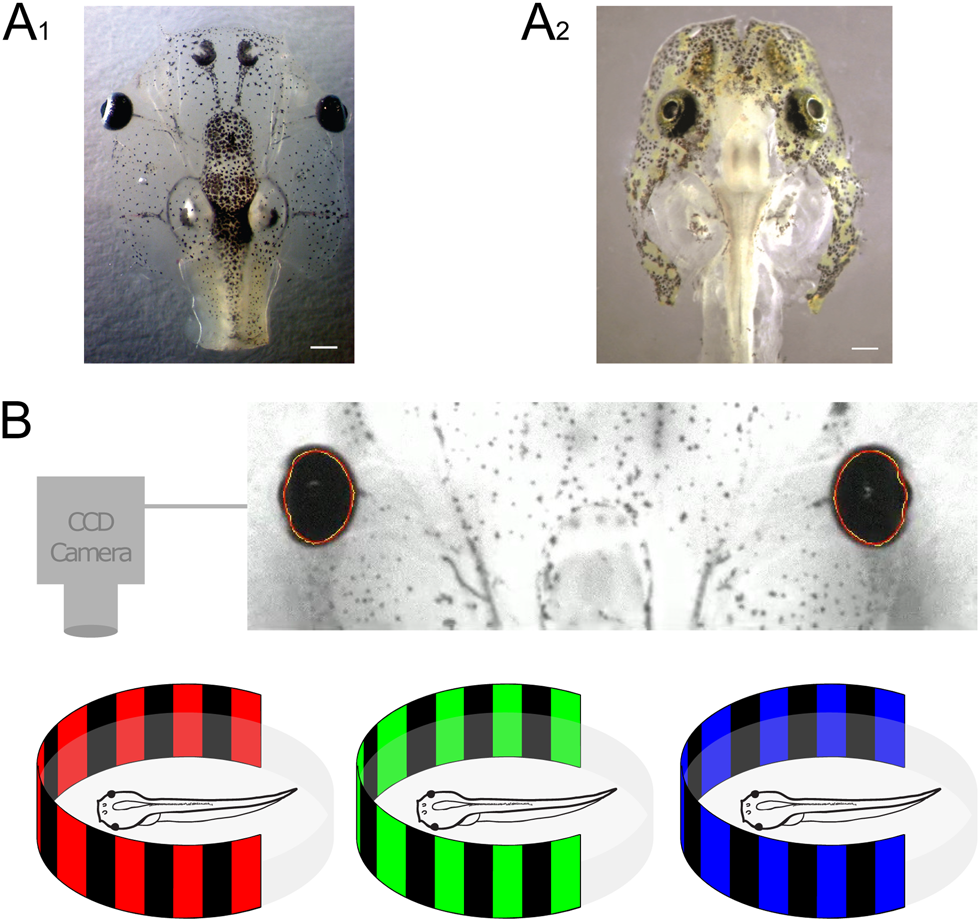
**Behavioral imaging of optokinetic responses in semi-intact amphibian preparations.** (A) Images of semi-intact preparations of larval *Xenopus*: A_1_ modified from Ramlochansingh et al. (2014) and Axolotl (A_2_) at stage 53, with intact eyes, eye muscles and central visuo-motor circuits. (B) Schematics of infrared video recordings of the movements of both eyes with a CCD camera during horizontally oscillating vertical color (red, green, blue)-striped patterns projected onto a cylindrical screen (bottom). Scale bars: 1 mm.

**Fig. 2.**
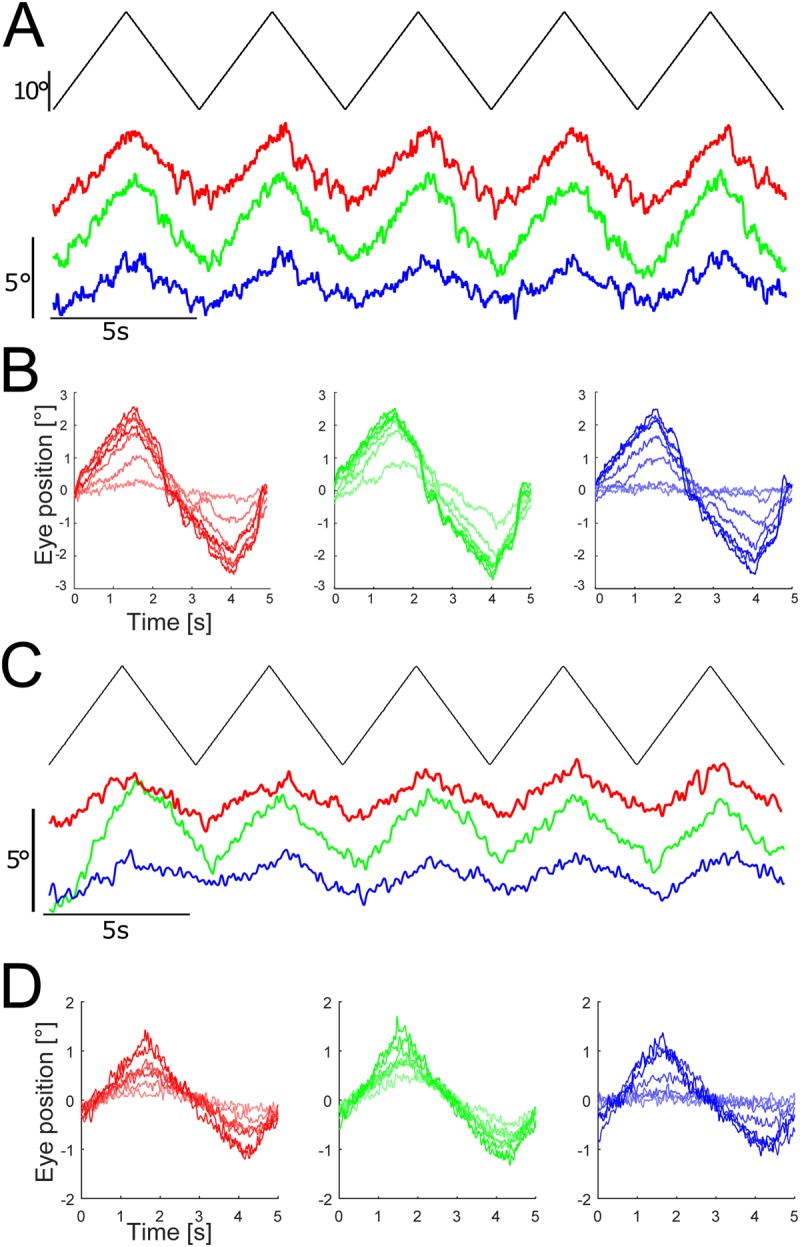
**Color-dependent optokinetic responses of *Xenopus* and Axolotl larvae.** (A,C) Representative examples of horizontal positional oscillations of the left eye (colored traces) extracted from video sequences during constant velocity oscillations (0.2 Hz; ±10°/s; black trace) of a large-field red-black, green-black and blue-black striped pattern (intensity level 32) in a *Xenopus* (A) and an Axolotl larvae (C). (B,D) Averaged responses over a single cycle during visual motion stimulation with different color-striped patterns (color coded) at seven selected luminance levels (intensity levels 8, 16, 32, 64, 96, 128, 255) in *Xenopus* (B) and Axolotl (D) obtained from the responses of the recordings depicted in A and C.

Large-field visual motion stimulation with black and white image patterns triggers robust ocular motor responses in semi-intact preparations of *X. laevis* tadpoles (Gravot et al., 2017). In a similar fashion, horizontally oscillating constant-velocity motion stimulation with a black and color (red, green, blue)-striped pattern (bottom schemes in Fig. 1B) elicited eye movements with comparable magnitudes in such semi-intact preparations of both *Xenopus* (Fig. 2A) and Axolotl larvae (Fig. 2C). With increasing stimulus intensity, the magnitude of eye movements increased gradually as indicated by the averaged responses over a single motion cycle (Fig. 2B,D). However, the amplitude of the eye movements at a particular luminance magnitude depended, in both species, on the color of the stripes (Fig. 2B,D). In order to calculate the perceptual sensitivity, the colored stripes were presented at 13 distinct intensities of increasing contrast (0, 4, 6, 8, 12, 16, 24, 32, 48, 64, 96, 128, 255) and the corresponding OKR amplitudes were analyzed. This allowed assessing the magnitude of the responses for each of the three primary RGB colors at increasing intensity levels in the two species, respectively.

**Fig. 3.**
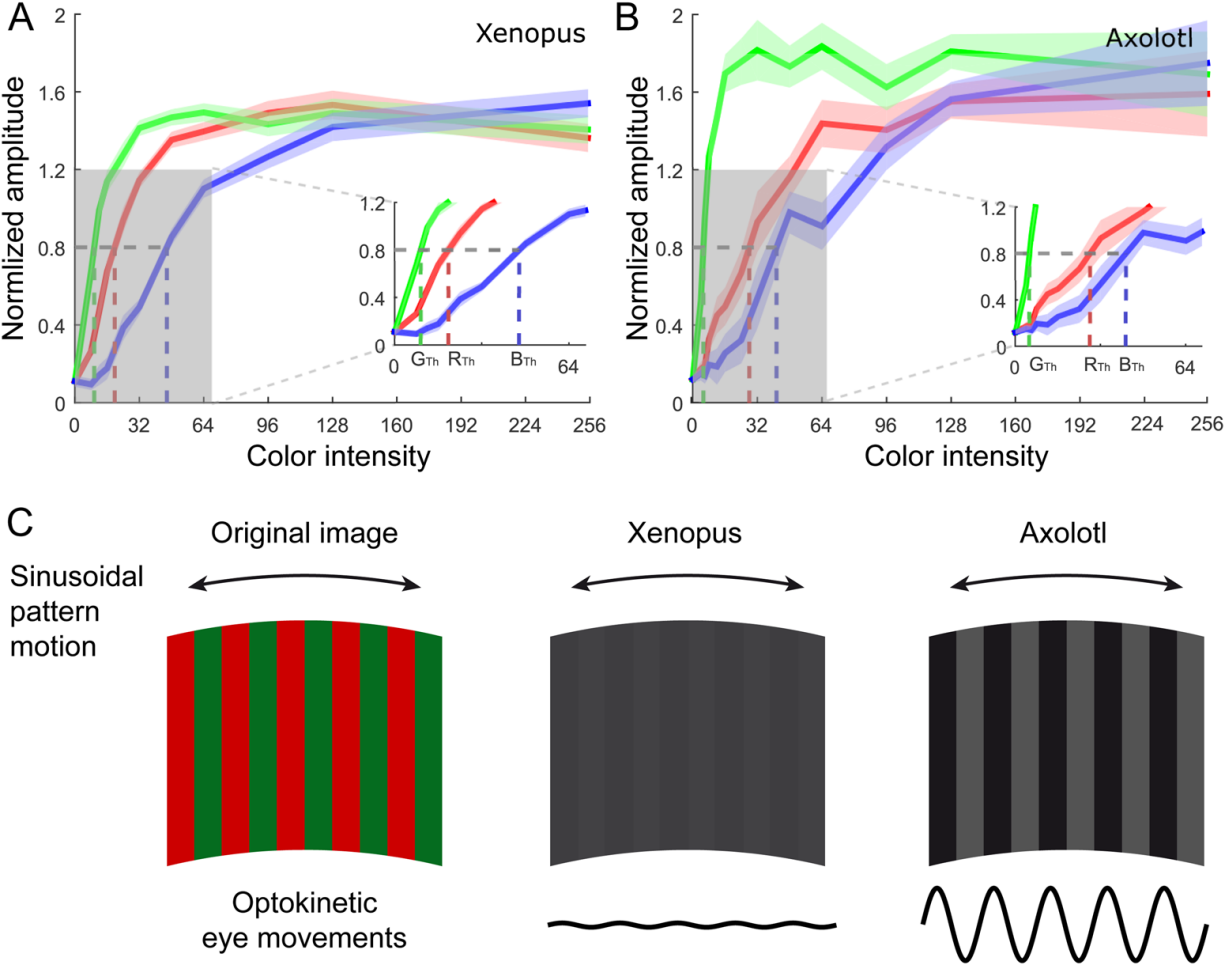
**Amplitude of optokinetic responses during motion stimulation with RGB colors.** (A,B) Dependency of normalized eye movement amplitudes during large-field image motion on the intensity of the three primary colors (RGB, color coded) in *Xenopus* (A, *n*=12) and Axolotl larvae (B, *n*=9). Bold lines represent the average and the shaded areas the standard error of the mean. The relative intensities required for an optokinetic response at the threshold level of 0.8 are denoted with R_Th_, G_Th_ and B_Th_ separately for the three component colors and shown at higher magnification in the insets in A,B. (C) Exemplary red/green-striped image motion pattern (left) and corresponding intensity contrast images that a *Xenopus* tadpole (middle) and an Axolotl larvae (right) theoretically perceive according to the different color sensitivities depicted in A,B; schematic eye movements (bottom) elicited by horizontal oscillating motion of the colored pattern (double-headed arrows) indicating that the same red/green-striped image evokes different eye motion magnitudes in the two species.

A differential sensitivity of the OKR amplitudes in larval *Xenopus* and Axolotl to the red, green and blue components of the striped large-field visual scene was systematically explored by comparing the stimulus intensities required to elicit an OKR with the same response amplitude of 80% of the mean amplitude across all stimuli (horizontal dashed lines at 0.8 in Fig. 3A,B), i.e. at approximately half of the saturation amplitude at maximal stimulus brightness (Fig. 3A,B). Linear interpolation allowed determining the intensities required for each of the three colors (R_Th_, G_Th_, B_Th_) to reach the same OKR amplitude. At all brightness levels below saturation, OKR magnitudes differed for red, green and blue color-striped patterns, however, differently in extent for *Xenopus* and Axolotl (see insets in Fig. 3A,B). In both species, patterns with green stripes evoked relatively large responses already at low luminance levels, followed by somewhat weaker responses to red stripes, while the smallest responses were encountered with blue stripes. This pattern was particularly prominent in Axolotl and less elaborate in *Xenopus*.

With respect to a response amplitude of 0.8, the relative weights for red, green and blue components, according to Eqn 3 were: c_R_=0.275, c_G_=0.585, c_B_=0.140 in *Xenopus* and c_R_=0.130, c_G_=0.782, c_B_=0.088 in the Axolotl. These estimates were very consistent between individual specimens and robust to variations in threshold. While the sensitivities determined for *Xenopus* tadpoles were surprisingly close to the reported values for human color perception (ITU-R BT.601-7: c_R_=0.299, c_G_=0.587, c_B_=0.114), Axolotl larvae showed a markedly higher relative sensitivity to the green channel, compared to red and blue color components. This difference is due to the wavelengths of the three primary RGB colors, which likely match less closely to the absorption spectra of the Axolotl photoreceptors involved in optokinetic behaviors than to those of *Xenopus*. If unaccounted for, these differences in spectral sensitivity have consequences for the interpretation of experimental data. Specifically, a moving color-image (left in Fig. 3C) that has clear brightness contrast for one species (right in Fig. 3C; Axolotl) is perceived as equiluminant by another species (middle in Fig. 3C; *Xenopus*). This difference would be accompanied by corresponding differences in the magnitude of many visuo-motor responses, such as optokinetic or optomotor reflexes (Krauss and Neumeyer, 2003) as well as neuronal activation of visuo-motor processing circuits, such as collision detector neurons in insects (Hartbauer, 2017). Indeed, optokinetic stimuli with a red-blue striped pattern at isoluminance (*R*/*B*=*c*_*R*_/*c*_*B*_) evoked only minimal ocular motor responses in *Xenopus* tadpoles. For a red-blue striped pattern with B=255, R=130, optokinetic response amplitudes diminished to 13%±10% (mean±s.d.) compared to unmatched stimuli, where the residual response and the variability can likely be attributed to inter-individual differences in the point of isoluminance.

In the examples described here, the physiological basis for using this relatively simple method to calibrate the species-specific color space is the extended functional integrity of semi-intact larval *Xenopus* and Axolotl (Straka and Simmers, 2012), and the robust expression of visuo- and vestibulo-motor reflexes in both species (Lambert et al., 2008; Direnberger et al., 2015; Gravot et al., 2017). The behavioral robustness of large-field visual image motion-evoked OKR in particular makes visuo-motor responses in such preparations excellently suitable for estimating how those animals perceive the brightness of primary colors in RGB displays for motion vision. The responses comply with the previously described requirements: there is a monotonous relation of image motion-evoked optokinetic responses with stimulus intensity (Gravot et al., 2017) and, like most low-level visuo-motor behaviors – there is a very weak response to pure color contrast at equiluminance (Neumeyer, 1998). Consequently, this simple behavioral assay is highly suitable to reveal species-specific differential sensitivities for the three primary colors of digitally produced images. Accordingly, our approach represents a simple and convenient method for investigating the perceived intensity of a visual stimulus in the RGB color space.

Based on the behavioral robustness, the characterization of RGB color sensitivity in both species yielded specific values that can be compared with those of other species including humans. Interestingly, the relative sensitivities to the component colors of a RGB display during optokinetic behavior of *Xenopus* tadpoles are remarkably similar to those used to calibrate monitors for human observers. This makes *Xenopus* tadpoles suitable as a viable model for studying color processing at subcortical visual levels in comparison to human subjects. More generally, our results demonstrate that the employed method gives consistent estimates of the spectral sensitivity between animals, is robust to the choice of the threshold level, and can successfully reveal species-specific differences in the perception of color presented with digital display devices in the RGB color space. Since biophysical and physiological processes of motion vision are treated as a ‘black box model’ described by Eqns 1 and 2, it is further possible to estimate the sensitivities to the primary colors using only the RGB values in the digital image as well as the behavioral readout, both of which are easily accessible by the experimenter. Therefore, it is not necessary to probe intermediate stages of the neuronal circuitry, thus eliminating the need to relate neuronal activity patterns to subjective perception. Instead, it draws upon a hard-wired behavioral response based on the intensity of the color image motion.

Since motion vision predominantly relies on pure luminance contrast (Reichardt, 1987), the method employed in the current study is limited to the estimation of the color sensitivity of a particular visuo-motor behavior that is elicited by activating respective sets of motion-sensitive retinal ganglion cells. Thus, the results presented here cannot provide an estimate about static color vision, i.e. whether and how animals discriminate between two distinct colors in the absence of motion (color contrast), but only allows drawing conclusions about the luminance contrast of a colored visual scene. Further limitations of the presented approach are related to the ‘black box’ approach: since all neuronal transduction processes are summarized empirically by the non-linear relation described in Eqn 2, it is impossible to identify a distinct substrate that is responsible for potential species-specific differences in color sensitivity. Further, it does not provide a full assessment of spectral sensitivity over the entire visible light spectrum. Nevertheless, since digital color reproduction is typically performed with RGB displays, knowledge about spectral sensitivities at wavelengths that are not emitted by the display is typically unnecessary. Thus, the determined sensitivities can be used to adequately tune VR setups in order to provide a species-specific and well-defined luminance contrast pattern of visual sceneries to the experimental animal. This is not only essential for eliciting appropriate natural behavioral responses, but also for distinguishing e.g. pure color cues from combined color and intensity cues in a virtual environment. In addition to the possibility to use the optokinetic reflex as a powerful tool for gaining insight into visuo-motor transformations (Benkner et al., 2013; Brockerhoff, 2006), many VR setups rely on other optomotor responses as behavioral measures for perception, such as body kinematics (Gray et al., 2002). Accordingly, our technique not only supplements other methods (e.g. Tedore and Johnsen, 2017), but also represents a procedure to easily and non-invasively obtain the required data in existing standard or adapted virtual reality environments. Further, it allows non-invasive measurements of the primary spectral sensitivity for each species, thus disclosing potential inter-individual and inter-species variations in color perception. In addition, our RGB calibration method can also be used as a rapid procedure to validate calibration parameters obtained with other methods (Tedore and Johnsen, 2017; Chouinard-Thuly et al., 2017).

## CONCLUSION

RGB color reproduction of digital display devices can provide accurate and well-defined contrast patterns for human observers. It is, however, unlikely that the human-adapted color space equally applies to other mammalian or non-mammalian species without further calibration. Here, we present a relatively simple method to estimate an animals' sensitivity to component colors of moving RGB images based on robust visuo-motor reflexes and demonstrate the applicability of this approach by revealing inter-species differences in the color sensitivity of optokinetic behaviors in *X. laevis* and *A. mexicanum* (Axolotl).

## MATERIAL AND METHODS

### Computation of RGB sensitivity

The relative perceived intensity *I* of any color (*R, G, B*) in the RGB color space can be computed as (ITU-R BT.601-7):(1)
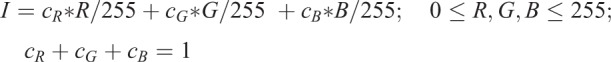
with c_R_, c_G_, c_B_ indicating the relative perceptual sensitivity of an evoked neuronal or behavioral response to the three component colors. These parameters, while known for humans (ITU-R BT.601-7), likely differ for other species, due to differences in retinal photoreceptor sensitivity. These parameters can be estimated from e.g. the performance of a visuo-motor behavior, in which the response amplitude *A* depends monotonously (though not necessarily linearly) on the perceived intensity *I* of the stimulus, which is the case for a large range of visuomotor behaviors studied over the past decades (e.g. Schaerer and Neumeyer, 1996; Rinner et al., 2005; Yamaguchi et al., 2008):(2)
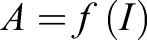
This function was sampled experimentally for the three primary colors, using visual stimuli in either color channel, while the other two channels were turned off. From the obtained curves, a constant threshold value for the response amplitude was chosen, and the required intensities of the red (R_Th_), green (G_Th_) and blue (B_Th_) stimuli to evoke the threshold amplitude were identified. Accordingly, the relative sensitivity of the visuo-motor behavior with respect to the individual primary colors was computed as:(3)
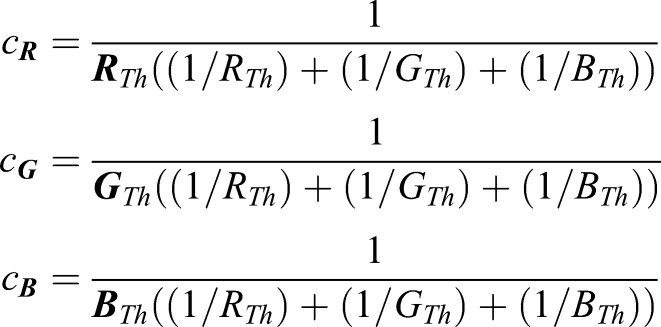


### Animals and experimental preparation

Larval *X. laevis* (*n*=12) at stage 53–54 (Nieuwkoop and Faber, 1994) and *A. mexicanum* (Axolotl; *n*=9) at stage 53–55 (Nye et al., 2003) of either sex were obtained from the in-house animal breeding facility at the Biocenter-Martinsried of the Ludwig-Maximilians-University Munich. Sample sizes were determined based on pilot experiments. Animals were maintained in tanks with non-chlorinated water (17–18°C) at a 12/12 light/dark cycle. Experiments were performed *in vitro* on isolated semi-intact preparations and comply with the ‘Principles of animal care’, publication No. 86–23, revised 1985 of the National Institute of Health. Permission for these experiments was granted by the liable authority at the Regierung von Oberbayern (55.2-1-54-2532.3-59-12).

Larvae of both species were anesthetized in 0.05% 3-aminobenzoic acid ethyl ester methanesulfonate (MS-222; Pharmaq Ltd. UK) in ice-cold frog Ringer solution (75 mM NaCl, 25 mM NaHCO_3_, 2 mM CaCl_2_, 2 mM KCl, 0.1 mM MgCl_2_, and 11 mM glucose, pH 7.4) and decapitated at the level of the upper spinal cord. The skin above the head was removed, the cartilaginous skull opened from dorsal and the forebrain disconnected (Gravot et al., 2017). The remaining central nervous system, including eyes, optic nerves, central visual relay areas and extraocular motoneuronal innervation of eye muscles were functionally preserved (Fig. 1A_1,2_). Following dissection, preparations were kept at 14°C for 3 h, allowing for a recovery from the anesthesia (Ramlochansingh et al., 2014). For all experiments, preparations were fixed with insect pins to the Sylgard floor in the center of a circular recording chamber (5 cm diameter) that was continuously superfused with oxygenated (Carbogen: 95% O_2_, 5% CO_2_) frog Ringer solution at a constant temperature of 17.0±0.1°C.

### Visual stimulation and eye motion recording

For visual stimulation and eye motion tracking, the recording chamber was affixed to the center of an open cylindrical screen, encompassing 275° with a diameter of 8 cm and a height of 5 cm (Fig. 1B). Three digital light processing (DLP) video projectors (Aiptek V60), installed in 90° angles to each other were fixed on the table surrounding the screen and projected the visual motion stimuli (Packer et al., 2001; Gravot et al., 2017) at a refresh rate of 60 Hz onto the screen (Fig. 1B). For eye motion recordings, a CCD camera (Grasshopper 0.3 MP Mono FireWire 1394b, PointGrey, Vancouver, Canada), mounted 20 cm above the center of the recording chamber, permitted on-line tracking of horizontal eye movements (top image in Fig. 1B) by custom-written software following a similar procedure as described by Beck et al. (2004). The position of both eyes was digitized at a sampling rate of 50 Hz and recorded along with the visual motion stimulus (Spike2 version 7.04, Cambridge Electronic Design Ltd., Cambridge, UK). The chamber was illuminated from above using 840 nm infrared light, a wavelength that has been shown to be invisible for *Xenopus* (Witkovsky, 2000). An infrared long-pass filter in the camera ensured selective transmission of infrared light and a high contrast of the eyes for motion tracking and online analysis of induced eye movements. The exact position of the visual stimulus and both eyes was read out in the data acquisition program Spike2 (Version 7.04, Cambridge Electronic Design).

### Data acquisition

Optokinetic responses were recorded for stimuli consisting of three different vertically striped patterns: red-black, green-black and blue-black (bottom scheme in Fig. 1B) at relative intensities of 0, 4, 6, 8, 12, 16, 24, 32, 48, 64, 96, 128, 255. The waveform of the motion had a rectangular profile with a constant velocity of ±10°/s and a frequency of 0.2 Hz (Fig. 2A). Eye position data were preprocessed by a Gaussian low-pass filter at a frequency of 5 Hz and segmented into individual stimulus cycles, excluding all cycles with eye motion peak velocities >50°/s, thereby discarding episodes of ocular motor behavior other than optokinetic slow phase responses (Lambert et al., 2012; Gravot et al., 2017). The amplitude of the optokinetic reflex was then computed from the evoked eye movements by fitting the dynamics of the stimulus profile to individual stimulus cycles and by subsequently calculating the median amplitude of all individual cycles. Since the motion profile was identical for all trials, this allowed a direct comparison of the magnitude of optokinetic eye movements evoked under different visual conditions.

## Supplementary Material

First Person interview
