# Supplementary material for: I spy with my little eye: a simple behavioral assay to test color sensitivity on digital displays
Source: Biol Open. 2018 Aug 20;7(10):bio035725. doi: 10.1242/bio.035725 (PMC6215414; doi:10.1242/bio.035725)
Supplement: First Person interview [file biolopen-7-035725-s1.pdf]

FIRST PERSON

First person – Alexander Knorr and Céline Gravot

First Person is a series of interviews with the first authors of a selection of papers published in Biology Open, helping early-career researchers promote themselves alongside their papers. Alexander Knorr and Céline Gravot are co-first authors on 'I spy with my little eye: a simple behavioral assay to test color sensitivity on digital displays', published in BiO. At the time the study was conducted, Céline Gravot was a research assistant at the Ludwig-Maximilians-Universität München, Germany, investigating how visual scene characteristics influence an animal's low-level internal representation of self- and world-motion. Alexander Knorr was a research assistant in the lab of Prof. Stefan Glasauer at the Center for Sensorimotor Research, University Hospital Großhadern, Germany, investigating how animals and humans build internal models of the world and use those models to optimize oculomotor behavior.

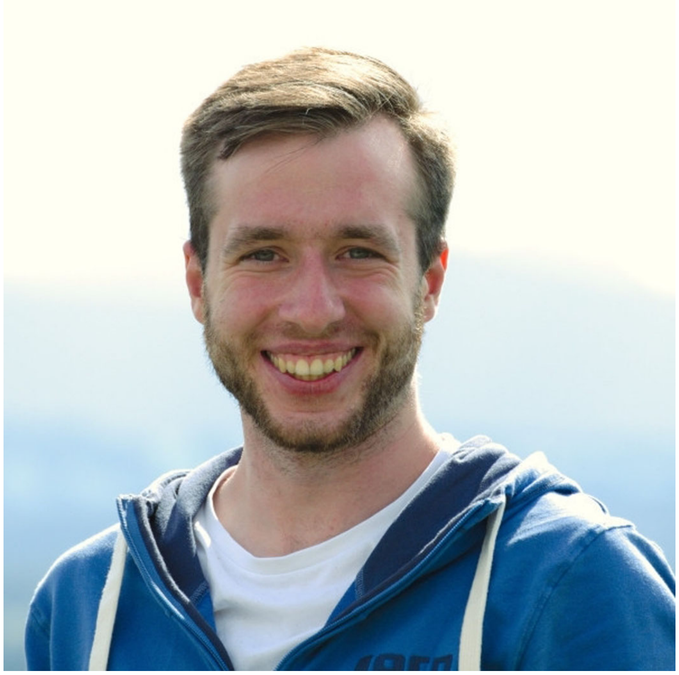

Alexander Knorr

What is your scientific background and the general focus of your lab?

Our respective backgrounds are neurobiology (Céline Gravot) and electrical engineering (Alexander Knorr), and we are working in a lab which is mostly concerned with research on the vestibular system and eye movements. This combination of experimental and technical expertise allowed us to establish a novel virtual reality setup in our laboratory which we then used to investigate the interaction of different modalities of (self-)motion perception, in particular the vestibular and visual motion systems.

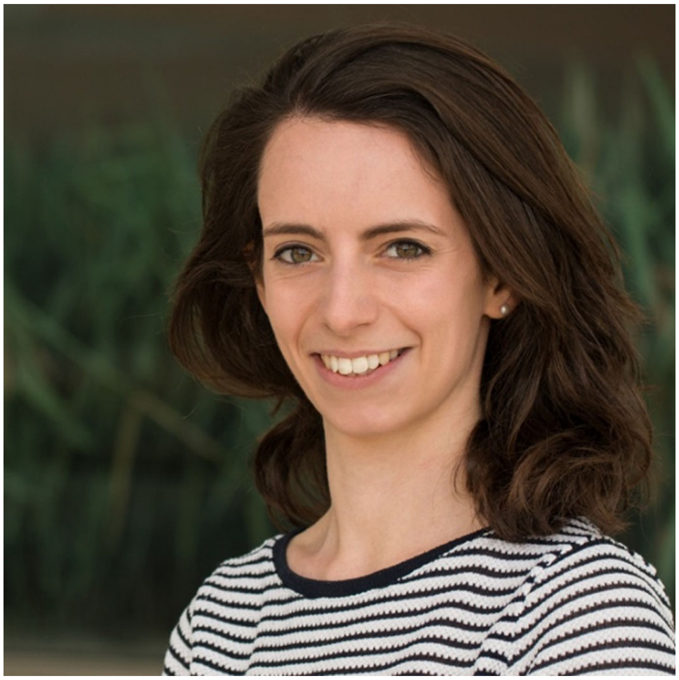

Céline Gravot

How would you explain the main findings of your paper to non-scientific family and friends?

Many researchers use digital displays (e.g. TV screens) for their animal experiments. These screens have, however, been built for the human eye. Animals perceive the world differently, so we don't know how animals see the images we show them in our experiments. Our article proposes a method which we can use to figure out how sensitive the animals are to the colors used in the displays. This gives us an idea of how the visual scenes that we present using video projectors in our experiments might look to them.

What are the potential implications of these results for your field of research?

With the rise of virtual reality (VR) technologies being used in neurobiology, we think that the method we describe will help other researchers using VRs to further improve the ecological validity of their experiments. Further, applying the described method to different species and using different types of behavior (e.g. eye movements, locomotion, etc.) might give insight into which types of retinal photoreceptors are involved in different visuomotor behaviors.

What has surprised you the most while conducting your research?

We found it quite surprising that the following reflex of *Xenopus laevis* tadpoles had very similar sensitivities to the different colors as

Alexander Knorr's contact details: Center for Sensorimotor Research, University Hospital Großhadern, Feodor-Lynen-Str. 19, 81377 München, Germany.
Céline Gravot's contact details: Department Biology II, Ludwig-Maximilians-Universität München, Großhaderner Str. 2, 82152 Planegg, Germany.
E-mails: Alexander.Knorr@lrz.uni-muenchen.de; gravot@bio.lmu.de

the human eye. We think that this property makes *Xenopus* tadpoles a very interesting animal model for studying visual perception on digital screens.

What, in your opinion, are some of the greatest achievements in your field and how has this influenced your research?

The use of virtual reality setups for animal models is a novel, but very promising avenue of research which we believe is just about to find its place in the field of neurobiology. Further developments in this direction will give us previously unimagined ways to investigate neuronal computations. We have taken this as our inspiration to suggest a method which helps to validate the use of digital displays in VR experiments on animals.

"Science should be a collaborative effort after all, and should be about gaining insight into nature, not about getting the next grant or publication."

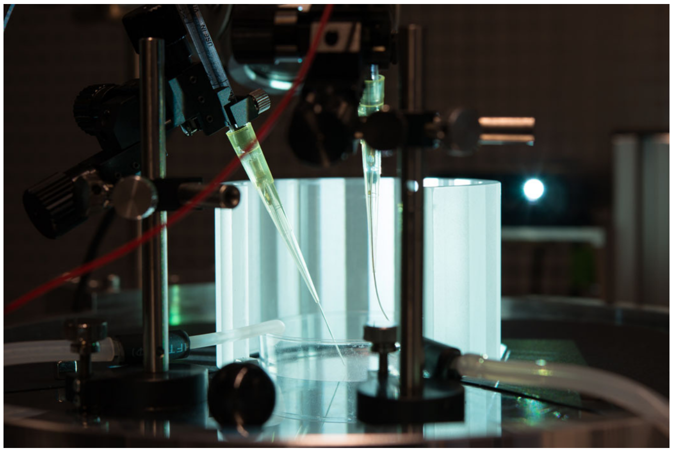

A strongly simplified visual scene (an optokinetic drum pattern) virtual reality setup, used to investigate how visual motion signals generate a self-motion perception in *Xenopus* tadpoles.

What changes do you think could improve the professional lives of early-career scientists?

One very important issue we and many of our colleagues face is the lack of a clear perspective in science after we finish our PhD. Many of us are very enthusiastic about our research, but when it comes to deciding for or against an academic career, the prospect of a long-term secure and well-paid job in industry, compared to going for a two-to-three year post-doc (and then struggling to find a tenured position afterwards), lead many of us to leave research. We realize that it would take major changes to the funding and organizational structure of academia to improve this, but we are convinced that many a highly competent and motivated young researcher would love to continue contributing to science and mankind's knowledge about nature and technology, without the risk of ending up unemployed at the age of 40, after the third post-doc without a permanent position in sight. On the other hand, one aspect that we feel is currently improving greatly is the trend towards open access in scientific publications, which we hope will reduce the competition between researchers to publish in high-impact journals. Science should be a collaborative effort after all, and should be about gaining insight into nature, not about getting the next grant or publication.

What's next for you?

Céline Gravot has recently graduated and is now working to communicate science to the public in the Deutsches Museum, one of the leading museums for technology and science in the world. Alexander Knorr is just about to graduate and will then seek a job in industry R&D which applies the research-driven approach of academia to satisfy the demands of an ever-evolving world.

Reference

Knorr, A. G., Gravot, C. M., Gordy, C., Glasauer, S. and Straka, H. (2018). I spy with my little eye: a simple behavioral assay to test color sensitivity on digital displays. *Biol. Open* **7**: bio035725.
